# Improving partnerships with family members of ICU patients: study protocol for a randomized controlled trial

**DOI:** 10.1186/s13063-017-2379-4

**Published:** 2018-01-04

**Authors:** Daren K. Heyland, Judy Davidson, Yoanna Skrobik, Amanda Roze des Ordons, Lauren J. Van Scoy, Andrew G. Day, Virginia Vandall-Walker, Andrea P. Marshall

**Affiliations:** 1 Department of Critical Care Medicine, Queen’s University, Kingston, ON Canada; 2 Clinical Evaluation Research Unit, Kingston General Hospital, Kingston, ON Canada; 3 EBP/Research Nurse Liaison, University of California, San Diego Health, San Diego, CA USA; 4 Department of Medicine, McGill University, Montreal, QC Canada; 5 Department of Critical Care Medicine and Division of Palliative Medicine, University of Calgary, Calgary, AB Canada; 6 Department of Medicine and Humanities, Division of Pulmonary, Allergy and Critical Care, Pennsylvania State University, Hershey, PA USA; 7 Faculty of Health Disciplines, Athabasca University, Athabasca, AB Canada; 8 Faculty of Nursing, University of Alberta, Edmonton, AB Canada; 9 Menzies Health Institute Queensland, Griffith University and Gold Coast Health, Southport, QLD Australia; 10 Kingston General Hospital, Angada 4, Kingston, ON K7L 2 V7 Canada

**Keywords:** Patient and family engagement, Randomized trial, Nutrition, End of life decision-making, Supportive care, Critical care

## Abstract

**Background:**

Over the last decade, health care delivery has shifted to partnering with patients and their families to improve health and quality of care, and to lower costs. Partnering with family members (FMs) of critically ill patients who lack capacity is particularly important for improving experiences and outcomes for both patients and FMs. How best to apply such partnering strategies, however, is yet unknown. The IMPACT trial will evaluate two interventions that enable partnerships with families of critically ill patients, each in a distinct content area, but similar in that they empower and support FMs.

**Methods:**

This multi-center, open-label, randomized, phase II clinical trial aims to randomize 150 older, long-stay ICU patients and their families into one of three groups (50 in each group): (1) The OPTimal nutrition by Informing and Capacitating FMs of best practices (OPTICs) group, a multi-faceted intervention to engage and empower FMs to advocate for, and audit, best nutritional practices for their critically ill FMs, (2) A web-based decision-support intervention called the ICU Workbook (The Canadian Researchers at the End of Life Network (CARENET) ICU Workbook; https://www.myicuguide.ca/. Accessed 3 Feb 2017.) to support families in shared decision-making process regarding goals of medical treatments, and (3) Usual care. The main outcomes for this trial include nutritional adequacy in hospital and hand-grip strength prior to hospital discharge; satisfaction with decision-making; decision conflict; and degree of shared decision-making.

**Discussion:**

With the goal of improving the functional recovery of nutritionally high-risk older patients and the quality of care at the end of life for these patients and their FMs in the ICU, we have proposed two novel family capacitation strategies. We hope that the nutrition and decision-support interventions implemented and evaluated in our study will contribute to the evidentiary basis for best family partnered care pathways focused on optimizing the quality of ICU care for patients with life-threatening illness and their families.

**Trial registration:**

Clinical trials.gov, ID: NCT02920086. Registered on 30 September 2016. Protocol version dated 11 October 2016.

**Electronic supplementary material:**

The online version of this article (doi:10.1186/s13063-017-2379-4) contains supplementary material, which is available to authorized users.

## Background

Over the last decade, health care delivery has shifted to partnering with patients and their families to improve health and quality of care, and to lower costs. Partnering with family members (FMs, i.e., immediate family, relatives, friends, and significant others) of critically ill patients who lack capacity is particularly important for improving experiences and outcomes for both patients and FMs [1–3]. Partnering with FMs decreases patient anxiety, confusion and agitation [4], reduces complications [5], decreases intensive care unit (ICU) and hospital length of stay [6], and improves long-term cognitive performance [7]. Overall, partnering with families helps patients feel more secure and increases patient and family member satisfaction [8–10]. Independent of its effect on patient outcomes, partnering with FMs has also been shown to reduce *their* anxiety, depression and psychological symptoms [5, 6, 11, 12]. Thus, such strategies that improve patient and FM outcomes, shorten ICU and hospital length of stay, and have the potential to save billions of dollars per year in health care costs [11].

How best to apply such partnering strategies, however, is unknown. The IMPACT trial will evaluate two interventions that enable partnerships with families of critically ill patients, each in a distinct content area, but similar in that they empower and support FMs. The first is a nutritional intervention, the *OPTimal nutrition by Informing and Capacitating family members of best practices (OPTICs)*, a multi-faceted intervention to engage and empower FMs to advocate for, and audit, best nutritional practices for their critically ill FMs. The second is a web-based decision-support intervention called the ICU Workbook [13] to support families in shared decision-making process regarding goals of medical treatments.

Herein, we describe the methodological approach to testing these interventions in the context of a phase II randomized clinical trial, the IMPACT trial.

## Methods

### Conceptual frameworks

**Fig. 1:**
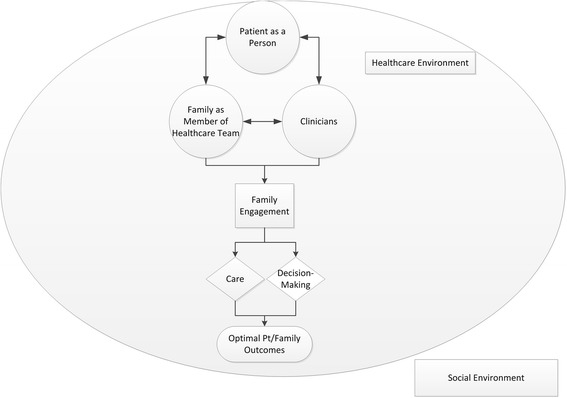
Conceptual model of family engagement in the intensive care unit (ICU). Many factors impact medical decisions for a patient. Patient factors include the patient as a person and their medical condition. Patient as a person represents the person’s prior experiences, values, preferences and goals. Environmental factors include the health care environment situated within a larger societal context. Family members may engage in care or decision-making. The family’s role in care gives purpose in crisis and may help family members cope with the exposure to critical illness. Their direct participation may also improve patient adherence to treatment plan and attainment of treatment goals. In shared decision-making the family is engaged as a member of the health care team. The family is typically most familiar with the patient as a person and the patient’s past health status. The clinicians are typically most knowledgeable of the patient’s critical illness. The decision-support intervention is designed to facilitate communication between the family and clinicians about the patient as a person and their medical condition. Family engagement in this manner facilitates a shared medical decision that is consistent with the patient’s values and goals in the context of their illness experience and medical condition, and is congruent with what the patient would choose if they were competent to make such a decision. Thus, we hypothesize that family engagement can influence family response to critical illness, and also the treatment plan. Ultimately, both patient and family outcomes are optimized

There are many determinants of the medical care that patients receive and their subsequent outcomes that include health systems and larger social factors (see Fig. 1). In an environment where the FM best knows the patient and the heath care team best knows the patient’s medical condition, we envision both coming together to make shared decisions and optimize patient outcomes. However, many FMs do not have the knowledge or confidence to perform this role [14]. Accordingly, we set out to develop interventions that support families in this key partnering role.

**Fig. 2:**
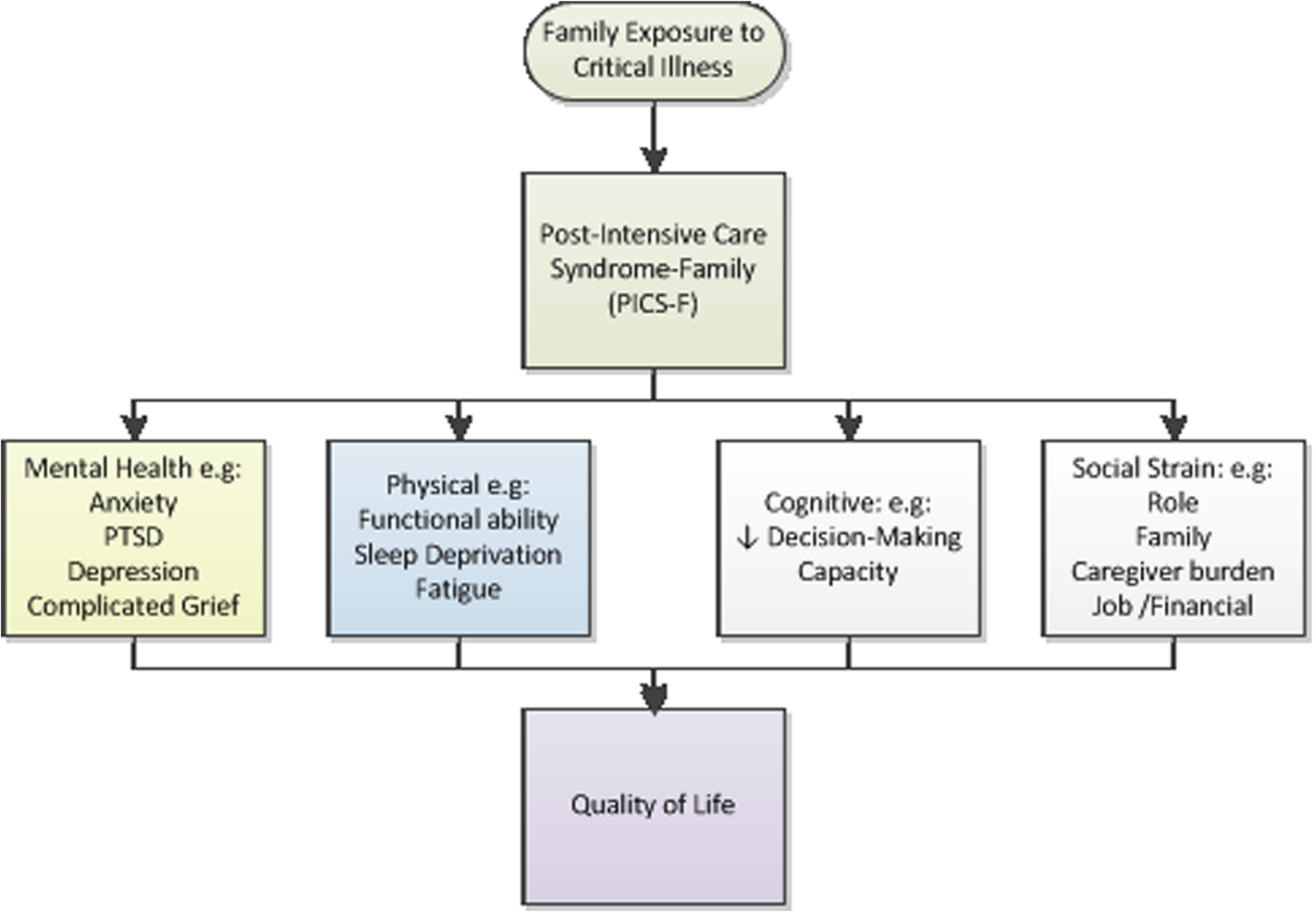
Post-intensive care syndrome among families of intensive care unit (ICU) survivors. Reprinted with permission from Springer [20]

To inform the development of the interventions, we used three theoretical frameworks (*Lightening our Load* [15], *Working to Get Through* [16], and *Facilitated Sensemaking* [17, 18]) developed by members of our team. These models recognize the importance of FM involvement and participation in communication, decision-making, and bedside care. The resultant sense of purpose and control may reduce post-intensive care syndrome, a series of stress-related complications experienced by FMs of critically ill patients (Fig. 2) [19, 20].

The background rationale and prior development work related to these interventions are discussed in Additional file 1.

### Overall aims and hypotheses

Our overall aim is to investigate whether, and how, the tools, knowledge, and skills provided to FMs will increase their satisfaction with care, and their sense of efficacy to act as advocates for best practice. We hypothesize that the multi-faceted strategies that engage families in patient care will: (1) increase the patient’s nutritional intake; (2) optimize physical recovery in older critically ill patients at high risk of nutritional problems; (3) reduce FMs’ psychological distress; (4) improve family satisfaction with decision-making; and (5) reduce the duration of ICU stay for future decedents. We further hypothesize that the trial will be feasible (as judged by enrollment rates and compliance with the protocol), the interventions efficacious, and contamination rates low (<10% of families in the usual care group will have been exposed to either or other interventions). The primary and secondary outcomes for each intervention are different and are explained below.

### Study design

**Fig. 3:**
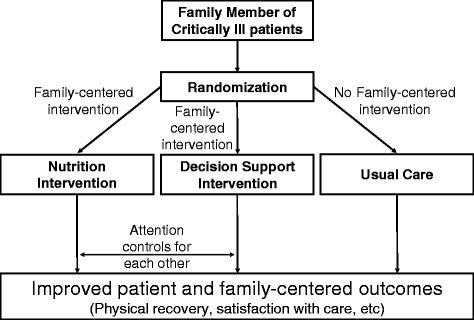
Study overview

This multi-center, open-label, randomized, phase II clinical trial involves three groups (two active interventions and one usual care, see Fig. 3). We report the methods of this study according to the Standard Protocol Items: Recommendations for Interventional Trials (SPIRIT) Checklist (see Additional file 2). By comparing the effects of the two treatment groups to the usual care group, we will understand the overall treatment effect of each intervention. To control for the possibility that the extra time and attention provided to families through the interventions may alter their perception of care and impact the outcomes of interest [21], we will also compare the treatment effects between two active interventions, thereby addressing this potential “placebo effect.” The comparison of the two active interventions groups will be considered secondary to the comparisons of each active group to the usual care group. All three comparisons will be pairwise with no pooling of groups.

### Setting

Eight tertiary ICUs in Canada (*n* = 4), the United States (*n* = 2), and Australia (*n* = 2) will participate in this phase II trial to understand important geographical, jurisdictional, or cultural factors that may influence the feasibility and efficacy at this stage of the program’s development. If the phase II trial is successful, the findings will be generalizable to a broader range of ICUs nationally and internationally.

### Study population

Given the nature of the study interventions, FMs of ICU patients who are “nutritionally high-risk” and/or those at risk of dying in the ICU, or during the subsequent hospitalization will be eligible to participate. The specific patient and FM eligibility criteria are presented in Additional file 3. Research coordinators (RCs) will review census lists and screen hospital charts daily to identify potentially eligible patients.

#### Randomization

Consent from eligible FMs will be obtained within 72 h following admission to the local ICU after the RC has explained the study objectives and procedures. Once informed consent is obtained, the RC will log onto the web-based randomization system. The randomization system will use a computer-generated randomization schedule allocating patients 1:1:1 by the method of permuted blocks of random undisclosed size within strata, to either (1) the OPTICs intervention, (2) the decision-support intervention, or (3) usual care. Randomization will be stratified by site.

#### Study interventions

**Fig. 4:**
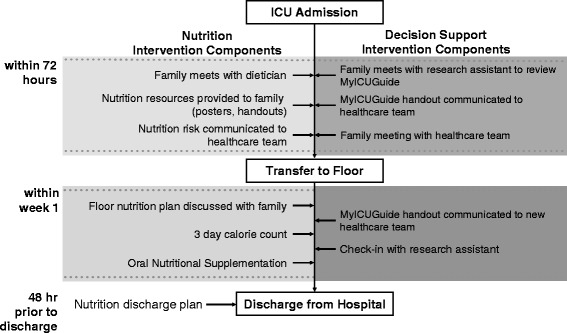
Timeline of the interventions

Following randomization, the RC will meet the FM, complete the baseline data collection (see Additional file 4) and initiate the study procedures (see Fig. 4), as described below.

##### OPTICs nutrition intervention

FMs randomized to the OPTICs nutrition intervention will meet with a dietitian early in the patient’s ICU stay (within 72 h of randomization). The dietitian will collect the patient’s brief nutritional history, verbally communicate the results of the nutritional risk assessment to the FM and clinical team, and place copies of the assessment in the hospital record. In addition, they will educate the FM about nutritional support in the ICU, focusing on capacitating all FMs to interact with health care providers (HCPs) and ask them about the nutrition that their critically ill relative is receiving. The FM will be provided with a booklet that reiterates the nutritional information and includes materials to help the family approach and ask questions of HCPs. The dietitian will assess comprehension using “talk-back” techniques [22]. In addition to the written resource, the FM will have ongoing access to videos that provide similar information as the booklet. Posters will be placed in the patient’s room with information about the OPTICs intervention (where permitted). The dietitian will communicate with the FM throughout the ICU stay to answer questions and identify unmet needs.

At or near the time of the patient’s ICU discharge, the dietician will provide further education based on the patient’s current nutritional status (i.e., receiving nutritional support, eating by mouth, swallowing difficulties), information about the nutrition that the patient can expect once on the ward, and will show the FM an educational video specific to nutrition after ICU discharge. As soon as the patient transitions to oral intake, the dietitian will introduce the FM to the nutrition diary, an oral intake audit tool to record the patient’s oral food intake at each meal. The patient will be followed by the ICU dietitian while in the ICU and by the ward dietitian once transferred.

Once patients in all groups are taking liquids by mouth, they will receive two or more Oral Nutritional Supplements (ONS) per day (approximately 400 kcal/day) as the existing academic literature suggests a positive impact on patient outcomes [23]. This is considered to be part of standard of care, but in this nutritional intervention arm the FM will be encouraged to advocate for the patient to receive them, coach the patient to take them as ordered, and monitor oral intake of the supplements using the nutrition diary. A nutrition care plan for the ward will be developed by the dietitian and communicated to the FM and patient. A 3-day calorie count will be completed weekly to capture calories and protein consumed from oral intake, including ONS, while the patient is on the ward until hospital discharge or for a maximum of 4 weeks, whichever comes first. Just prior to hospital discharge, the dietitian will work with the patient and FM to develop a home nutrition plan provided to them in writing at the time of discharge. All study materials for the OPTICs intervention can be found on the Critical Care Nutrition website [24]. All other co-interventions will be permitted; this family engagement strategy will be provided in addition to usual care.

##### Decision-support intervention

The decision-support intervention consists of an online resource entitled “ICU Workbook” [13] that provides information to families on principles of shared decision-making, experiences that may be expected during the ICU stay, and advice on coping with the stress of having a FM in the ICU. This resource was developed from 19 qualitative interviews of FMs’ perceived stressors and coping strategies used during the decision-making process, and has been pilot tested [25]. Integrated into this online resource are several sections and resources listed in Table 1. The RC will go through the content of the website guide, using talk-back techniques to ensure that FMs understand the key concepts presented on the website [22]. Paper copies of the electronic content will also be given to FMs and a link to the website so they can return to review materials at a later date. After completing the ICU Workbook with FMs, the RC will verbally communicate the results of the exercises to the clinical team. The questions and answers will be summarized and copies of the report placed on the hospital record, and a copy will be given to the FMs. Next, the RC will work with the health care team to arrange a family meeting including the attending physician and bedside nurse to review patient goals of care within 72 h of completing the ICU Workbook. The RC will attend this meeting to record its content using the Observing Patient Involvement (OPTION) tool (described below). The RC will connect with FMs throughout the ICU stay and upon discharge from ICU, to build and maintain the relationship and answer questions, and to facilitate communication of the ICU Workbook report to the attending physician and clinical team on the ward.

**Table 1:** Components of the ICU Workbook – the decision-support intervention

Section	Description
1. Orientation and education about the ICU	• Provides a general overview of the intensive care unit (ICU) including key ICU terms, treatments, and roles of various clinicians who work in the ICU. • Describes common processes in the ICU, including resuscitation and comfort measures, organ donation, Power of Attorney, and substitute decision-making (SDM) • Defines commonly used vocabulary within the ICU.
2. When a loved one is in the ICU	• Provides suggestions for coping strategies for family members of an ICU patient. • Encourages family member visitation. • Offers advice about how to ask questions in the ICU. • Encourages family to seek support or keep a journal during the ICU stay.
3. Looking after yourself	• Reinforces the importance of self-care for families of ICU patients. • Encourages family members to sleep, eat, and maintain healthy physical activity • Provides ideas for how to inform and communicate with other family and friends about the patient’s progress.
4. Making decisions in the ICU	• Describes and encourages shared decision-making. • Defines the role and responsibilities of family members in SDM. • Defines the roles of clinicians in decision-making. • Provides resources available to help with the decision-making process.
5. Help us to get to know you and your family member	• Family directed questionnaire asking information about the patient’s personal characteristics. • Assesses family members’ state of mind and emotional status. • Assesses patient’s clinical status and frailty
6. Informational preferences	• Identifies family member’s desire for information and level of health literacy.
7. Values history tool	• Assesses patients’ values and preferences (as reported by family members). • Questionnaire helps family members articulate patient’s view of quality of life, value conflicts, impact of decisions on others, and religious/spiritual/cultural beliefs.
8. Decision preferences	• Elicits family member’s preferences for extent of information sharing and preferred role in decision-making • Identifies others who should be involved in decision-making • Measures residual decisional conflict

##### Usual care

Patients randomized to usual care will not receive any study interventions. Baseline data collection and outcome assessment will be performed similarly as for the intervention groups. Local practices will determine the extent to which families receive support from allied health care professionals in the management of nutrition and decision-making for their relative.

### Outcomes

The current project is a phase II trial focusing on short-term efficacy and feasibility outcomes. The future phase III IMPACT trial will likely have two co-primary outcomes related to the nutritional and decision-support interventions. For the nutritional intervention, the primary outcome will represent the patient’s physical recovery long term (i.e., 6-minute walk distance at or before hospital discharge, activities of daily living, and the 36-item Short Form survey (SF-36) Physical Function at 6 months) as suggested by current experts [26]. Secondary outcomes include adequacy of nutrition in the ICU, ONS consumption on the wards, time to discharge alive from hospital (time-to-event analysis with death as competing risk), ICU and hospital outcomes (mortality and length of stay), 90-day readmission rates, and the cost-effectiveness of the intervention. We expect that as a consequence of their involvement and engagement in patient care, FMs’ psychological well-being will be improved as well in this group. For the decision-support intervention, the primary outcome will be a measure of family psychological well-being (symptoms of depression and anxiety (Hospital Anxiety and Depression Scale [27]), and post-traumatic stress disorder (Impact of Events-Revised [28]) 6 months after the ICU stay). Secondary outcomes will include family satisfaction with decision-making using the Decision-making component of the Family Satisfaction with ICU Care (FS-ICU24) subscale and length of ICU stay for decedents (a marker of poor quality end-of-life (EOL) care from patients’ perspective and that has been responsive to prior palliative care interventions in the ICU).

For the purpose of this phase II study, we are evaluating more proximal (short-term) outcomes and process measures. These outcomes will be collected by the RC and will be assessed 2–3 weeks after ICU-related death or discharge or prior to hospital discharge, whichever comes first. Table 2 presents all process measures (short-term or long-term) planned for the current phase II and future III studies. To enable comparisons across all three groups, all process and outcome measures will be performed for all patients. With respect to the nutritional intervention, one of the key short-term outcomes will be nutritional adequacy during ICU stay. We have previously shown that patients who receive greater nutritional adequacy support in the ICU have better long-term outcomes [29]. In the ICU, to assess nutritional adequacy, the total amount of energy or protein received from either enteral nutrition (EN) or parenteral nutrition (PN), inclusive of propofol, will be divided by the amount prescribed in the baseline assessment and expressed as a percentage for patients in all groups. Unfortunately, no easy method exists to measure nutritional adequacy on the hospital wards where patients are likely taking nutrition by mouth. We plan to do 3-day calorie counts weekly for 4 weeks during the hospital study as well as collect the use of ONS from all study patients.

**Table 2:** Outcomes considered in the IMPACT protocol program of research

	Process measures	Short-term (hospital) outcomes^a^ (current phase II)	Long-term (months following hospital discharge) outcomes (future phase III)
Nutritional intervention	Compliance with the intervention Met with dietitian early in ICU stay and at ICU discharge to review materials Received a nutrition plan at ICU and ward discharge Used nutrition audit tool	Nutritional adequacy during ICU stay Consumption of Oral Nutritional Supplements during hospital stay Intake on hospital wards (3-day calorie count) Hand-grip strength at or before hospital discharge	Measures of patient’s long-term physical recovery (e.g., 6 MWD at 3 and 6 months)
Decision-support intervention	Compliance with the intervention Met with RC early and went through the myicuguide website Family meeting to review intervention output within 72 h of encounter Patient-centered report from MyICUGuide on chart during ICU and hospital stay and attending physician aware of contents of this report	Use of shared-decision making (OPTION^a^ tool) during initial family conference Change in decisional conflict at 1 week Family satisfaction with decision-making at ICU discharge (or 2–3 weeks later for decedents)	Measure of family member’s long-term psychological well-being Length of ICU and hospital stay for decedents
Other	Contamination (families exposed to an intervention to which they were not assigned) Exposed to myicuguide website Exposed to OPTICs content	Overall family satisfaction with ICU care at ICU discharge (or 2–3 weeks later for decedents)	

Table of current and projected outcomes for the IMPACT program of research

^a^All outcomes listed in this column will be measured in all randomized patients

6MWD, 6-minute walk distance, *ICU* intensive care unit

For the purposes of this phase II trial, we will assess physical recovery by measuring hand-grip strength at hospital discharge for patients in all groups. Impaired muscle strength is an important outcome because of its association with disability and functional decline, and reduced health-related quality of life (QOL) [30]. The OPTICs intervention FMs who actively engage with nutritional care of their loved ones may show improved family satisfaction with care. Additionally, engagement in care is theorized to support FM participation in patient ICU care [16, 18] and to be protective of the FM’s mental health by providing a sense of purpose in crisis [17].

We will evaluate the impact of the decision-support intervention on shared decision-making with FMs using the Observing Patient Involvement (OPTION) instrument, developed to evaluate communication during shared decision-making [31]. During the initial family conference after the decision-support intervention (or similar time frame for other groups), the RC will identify key shared decision-making behaviors (such as “did the clinician present pros and cons of various treatment options?”) and select a response from the 5-point scale ranging from “is not observed” to “observed and executed to a high standard.” The total summed score ranges from 0 to 48, with higher scores indicating greater competency in shared decision-making [31].

To assess the impact of the decision-support intervention on FM decision conflict, we will use the 10-item Decisional Conflict Scale (DCS) [32]. This scale is validated and reliable, and used extensively to evaluate the effectiveness of shared decision-making interventions such as patient decisions aids and counseling [33]. Scores range from 0 (no conflict) to 100 (high conflict); scores greater than 50 are associated with delayed decisions. This scale has to be linked to a decision made or an expressed preference. Accordingly, at baseline and at the end of the first week, we will elicit a preference for use of life-sustaining treatments in the ICU setting and administer the DCS to all participating FMs. At the same two time points, we will ask the FM about their preference for use of life-sustaining therapies.

We will also use the FS-ICU24 survey to obtain ratings of satisfaction with ICU care from designated FMs of all patients enrolled in the study. This questionnaire has been shown to have content and construct validity, high reliability (correlation coefficient = 0.85), and two validated subscales (Satisfaction with Overall Care and Satisfaction with Decision-Making) [34, 35]. We will administer FS-ICU24 to FMs of surviving patients upon ICU discharge and mail the questionnaire to FMs of decedents 2–3 weeks following death [34].

For this phase II trial, additional outcomes include the feasibility and fidelity of the implementation and measures of contamination as shown in Table 3. Plans to manage the data will be consistent with standard operating procedures at the coordinating center.

**Table 3:** Additional outcomes for the IMPACT trial

Parameter	Defined as…
Enrollment and consent rate	We will judge the current study protocol feasible if > 75% of eligible families of eligible patients are approached and > 60% of these consent.
Compliance with the components of the OPTICs intervention.	The dietitian will keep a log of all FMs with whom the OPTICs intervention materials were reviewed, the time the intervention was delivered, and whether a nutrition plan was presented at the end of the ICU stay (survivors only) and ward stay. At baseline, the review of these materials should occur in > 90% of enrolled FMs and the nutrition plan should be presented in > 75% of eligible cases for this to be considered feasible.
Compliance with the components of the family directed decision-support intervention	Review of the website should occur in 100% of the FMs enrolled in this group, and the family meeting (including the enrolled FM) should occur within 72 h in > 75% of cases for this intervention to be considered feasible. In addition, the RC will perform a chart review after ICU death or discharge for all enrolled patients and document evidence that the components of the decision-support intervention were included in the medical record. We will consider the protocol successful if > 75% of charts contained such evidence.
Physician Awareness Assessment	One week after enrollment, the RC will administer the Physician’s Awareness Assessment to the attending physician and/or fellow responsible for the care of the patient during the period of enrollment to assess the extent to which they were aware of study materials, the variables captured in the study intervention output (nutritional history, patient pre-morbid functional state, values, preferences, etc.) and the degree to which this knowledge influenced their decision-making. If > 75% of them acknowledge exposure to study tools and rate their impact as substantial, in the respective interventional groups, the intervention will be considered feasible.
Contamination	We will ask all FMs whether they have had a facilitated review of the myicuguide website and OPTICs tools. If < 10% of the families of patients in the usual care group acknowledge that they have seen the study tools and if < 10% of the intervention groups acknowledge they have been exposed to the other intervention, we will consider this acceptable.

*FM* family member, *RC* research coordinator, *ICU* intensive care unit

### Sample size

The total sample size for this phase II trial is 150 patients (50 per group). We expect the subsequent phase III trial will require 20–30 sites with up to 1000 patients in total. As one of the primary goals of this phase II trial is to assess the feasibility of implementing the study interventions, we plan to assess feasibility at multiple sites. With approximately 10–20 patients per site, we expect to gain enough experience to assess the feasibility of the study protocol per site.

For each intervention, we have performed a sample size calculation for one of the key short-term outcomes of each intervention. From our prior work, we know that the average nutritional adequacy of these patients during their ICU stay will be 40–50% with a standard deviation of 30% [36]. We aim to detect a small but clinically meaningful increase in nutritional adequacy of approximately 20%. Under these assumptions 50 patients per group will achieve 92% power at a two-sided alpha = 0.05. For the decision-support intervention, we will power the trial to evaluate family satisfaction with decision-making. In our prior REALISTIC-80 study, the standard deviation of the FS-ICU24 “Decision-making” domain was 11; we consider an increase of 5.5 points (a medium effect size) to be plausible and clinically important. With 50 evaluable subjects per group, we would achieve 71% power at a two-sided alpha = 0.05 to detect a 5.5-point difference between groups.

Given the other objectives of the study related to feasibility, compliance, and contamination, a sample size of 50 per group will allow us to assess these endpoints with reasonable precision, consistent with the sample size of other phase II studies. For example, with 50 patients per group there is a 95% chance of estimating a binary variable (such as loss to follow-up, contamination, compliance, etc.) to within ± 14% and any variable with a rate < 15% or > 85% (as would be expected for loss to follow-up, contamination, or compliance) could be estimated to within ± 10% or 19 times out of 20.

### Statistical analysis

The feasibility outcomes will be described by group as rates with 95% confidence intervals. Reasons for loss-to-follow-up, non-compliance, and contamination will be tabulated. The distribution of the continuous outcomes described above will be described by group and compared among groups using a mixed-effects model with treatment arm as a fixed effect and site as a random effect. Due to the limited (six to eight) number of sites in this phase II trial, we will perform a sensitivity analysis, treating site as a fixed effect. For the binary outcomes, we will use the Mantel-Haenszel test stratified by site. At this exploratory phase II stage we will not formally adjust *p* values for multiplicity of tests but will consider the potential type I and type II errors in our interpretation of results. For key efficacy outcomes with > 5% missing data, multiple imputation will be used for the primary analysis supplemented by a complete case sensitivity analyses. The heterogeneity between sites will be estimated by the intra-class correlation coefficient for all outcomes; in case this is needed to inform the design of a subsequent cluster randomized controlled trial (RCT) [37].

### Ethics

We will obtain local ethics approval at each participating site before commencing. RCs will obtain written informed consent from FMs for their participation in the trial and proxy consent from the same FM to enable data collection related to the patient. For surviving patients, as they regain competency, we will consent them prospectively for their continued involvement in the study (obtaining hand-grip strength prior to hospital discharge). Given that the interventions are not directed towards the patient (we are only collecting data from the hospital record), we will not be collecting or reporting serious adverse events and there will be no Data Monitoring Committee for this trial. There are no financial or other competing interests for any of the co-authors. Results will be published and posted on the study team’s internationally renowned websites [38, 39] to aid in dissemination.

## Discussion

With the goal of improving the functional recovery of nutritionally high-risk older patients and the quality of care by patients at the end of life and their FMs in the ICU, we have proposed two novel family capacitation strategies. We hope that the nutrition and decision-support interventions implemented and evaluated in our study will contribute to the evidentiary basis for a family partnered care pathway focused on optimizing the quality of ICU care for patients with life-threatening illness and their families. We aim to start enrollment in the first quarter of 2017.

## Additional files

Additional file 1: Description of background rationale for the OPTICs and decision-support intervention. (DOCX 47 kb)

Additional file 2: SPIRIT 2013 Checklist: recommended items to address in a clinical trial protocol and related documents. (DOCX 74 kb)

Additional file 3: Eligibility criteria. (DOCX 45 kb)

Additional file 4: Baseline data collection. (DOCX 45 kb)
