# Supplementary material for: Improving partnerships with family members of ICU patients: study protocol for a randomized controlled trial
Source: Trials. 2018 Jan 4;19:3. doi: 10.1186/s13063-017-2379-4 (PMC5753514; doi:10.1186/s13063-017-2379-4)
Supplement: Supplementary file 1 — Description of background rationale for the OPTICs and decision-support intervention. (DOCX 47 kb) [file 13063_2017_2379_MOESM1_ESM.docx]

**Additional file 1: Description of Background Rationale for the OPTICs and Decision Support Intervention**

*OPTimal Nutrition by Informing and Capacitating Family Members of Best Practices (OPTICS Intervention)*

Prior large-scale studies of critically ill patients have demonstrated that optimal amounts and timely provision of nutrition is associated with reduced infectious complications, duration of mechanical ventilation and mortality [1-3], and perceptions of more rapid physical recovery [4]. The treatment effect is greatest in patients at high nutrition risk [5]. Gaps in hospital processes of care (limited mobilization, inadequate nutritional intake, suboptimal continence care) account for the majority of patient disability observed after hospitalization [6]. Modifying these practices, and in particular increasing the provision of macronutrients, may improve the recovery and functional status of critically ill older patients and lessen the burden on families. Since repeated, sustained efforts over the past few years have not significantly improved the total amount of calories and protein delivered via the enteral route [7], innovative and multifaceted approaches are warranted. We propose a novel family engagement strategy, previously piloted for feasibility and acceptability [8,9], that empowers family members (FMs) to advocate for best nutrition practices, audit current nutrition practices, and facilitate the use of a specialized oral nutrition supplement (ONS) in critically ill, nutritionally high risk patients.

*The ICU Workbook Decision Support Intervention (MyICUGuide)*

Our previous research has shown that very elderly patients admitted to the intensive care unit (ICU) have a relatively low probability of surviving and returning to independent physical functioning one year after ICU admission [10]. One quarter of these patients will die in hospital, many after a prolonged ICU stay [6]. Another quarter will recover only to a state of significantly impaired function [6]. In addition to these poor outcomes, critical care itself is associated with pain, discomfort, and other distressing symptoms for the patient, psychological stress for FMs, moral distress for healthcare workers, and high costs. Moreover, treatment provided is often inconsistent with patient and family values and preferences for end of life (EOL) care [11]. To address this mismatch and the underlying deficiencies in communication and decision-making [12], our multifaceted intervention is designed to improve EOL communication and decision-making in very elderly patients admitted to the ICU with life-threatening illnesses. The decision support intervention is feasible and acceptable in pilot-testing with 27 families of ICU patients whose feedback was used to refine the intervention [13].

**References**

[1] Alberda C, Gramlich L, Jones N, Jeejeebhoy K, Day AG, Dhaliwal R, et al. The relationship between nutritional intake and clinical outcomes in critically ill patients: results of an international multicenter observational study. Intensive Care Med. 2009;35:1728-37.

[2] Heyland DK, Stephens KE, Day AG, McClave SA. The success of enteral nutrition and ICU-acquired infections: a multicenter observational study. Clin Nutr. 2011;30:148-55.

[3] Heyland DK, Cahill N, Day AG. Optimal amount of calories for critically ill patients: depends on how you slice the cake! Crit Care Med. 2011;39:2619-26.

[4] Zigmond AS, Snaith RP. The hospital anxiety and depression scale. Acta Psychiatr Scand. 1983;67:361-70.

[5] Heyland DK, Dhaliwal R, Jiang X, Day AG. Identifying critically ill patients who benefit the most from nutrition therapy: the development and initial validation of a novel risk assessment tool. Crit Care. 2011;15:R268.

[6] Zisberg A, Shadmi E, Gur-Yaish N, Tonkikh O, Sinoff G. Hospital-associated functional decline: the role of hospitalization processes beyond individual risk factors. J Am Geriatr Soc. 2015;63:55-62.

[7] Heyland DK, Dhaliwal R, Wang M, Day AG. The prevalence of iatrogenic underfeeding in the nutritionally 'at-risk' critically ill patient: Results of an international, multicenter, prospective study. Clin Nutr. 2015;34:659-66.

[8] Marshall AP, Wake E, Weisbrodt L, Dhaliwal R, Spencer A, Heyland DK. A multi-faceted, family-centred nutrition intervention to optimise nutrition intake of critically ill patients: The OPTICS feasibility study. Aust Crit Care. 2016;29:68-76.

[9] Heyland DK, Lemieux M, Dhaliwal R, Seyler H, MacEachern K, Marshall AP. Optimal Nutrition by Informing and Capacitating Family Members of Best Practices: The OPTICS feasibility study. ASPEN Clinical Nutrition Week 2016, Austin, Texas.

[10] Heyland DK, Garland A, Bagshaw SM, Cook D, Rockwood K, Stelfox HT, et al. Recovery after critical illness in patients aged 80 years or older: a multi-center prospective observational cohort study. Intensive Care Med. 2015;41:1911-20.

[11] Heyland DK, Barwich D, Pichora D, Dodek P, Lamontagne F, You JJ, et al. Failure to engage hospitalized elderly patients and their families in advance care planning. JAMA Intern Med. 2013;173:778-87.

[12] Heyland DK, Dodek P, Mehta S, Cook D, Garland A, Stelfox HT, et al. Admission of the very elderly to the intensive care unit: family members' perspectives on clinical decision-making from a multicenter cohort study. Palliat Med. 2015;29:324-35.

[13] Chiarolanzio PJ, Kraft J, Reading J, Van Scoy LJ, Heyland D. Decisional Support Guide for Families of ICU Patients: A Multicenter Feasibility Study. Am J Respir Crit Care. 2016;C26:4742-A4742.
