# Supplementary material for: Improving partnerships with family members of ICU patients: study protocol for a randomized controlled trial
Source: Trials. 2018 Jan 4;19:3. doi: 10.1186/s13063-017-2379-4 (PMC5753514; doi:10.1186/s13063-017-2379-4)
Supplement: Supplementary file 3 — Eligibility criteria. (DOCX 45 kb) [file 13063_2017_2379_MOESM3_ESM.docx]

**Additional file 3: Eligibility Criteria**

Given the nature of the study interventions, we are targeting ICU patients who are ‘nutritionally high-risk’ and/or those at risk of dying in the ICU or the subsequent hospitalization. For patients to be considered eligible, they must be:

1a) ≥ 60 years of age

OR

1b) 55 years to 59 years old with one or more of the following diagnoses:

- *Chronic obstructive lung disease* - 2 of the 4 of: baseline PaCO2 of > 45 torr, cor pulmonale; respiratory failure episode within the preceding year; forced expiratory volume in 1 sec <0.5 L.
- *Congestive heart failure* - New York Heart Association class IV symptoms and left ventricular ejection fraction < 25%.
- *Cirrhosis* - confirmed by imaging studies or documentation of esophageal varices and one of three conditions: a) hepatic coma, b) Child’s class C liver disease, or c) Child’s class B liver disease with gastrointestinal bleeding.
- *Cancer -* metastatic cancer or stage IV lymphoma.
- *End-stage dementia* (inability to perform all ADLs, mutism or minimal verbal output secondary to dementia, bed-bound state prior to acute illness)

AND

2) Have a projected duration of ICU dependency of >72 hours from time of final assessment. We define ICU dependency as the need for:

- Mechanical ventilation
- Non-invasive ventilation
- Renal replacement therapy
- Vasopressors or
- Artificial nutrition because of their underlying illness

(not patients that stay in ICU because of lack of bed availability)

We will exclude the following patients:

1. Patients who are not expected to remain in the ICU and alive for 72 hours after initial screening (physician judgment) or for whom life-sustaining treatments are expected to be withdrawn in the subsequent 72 hours (as sufficient time will be required for implementation of the study interventions).
2. Uncomplicated elective surgical patients (regardless of age)
3. Patients receiving long-term tube feeding pre-admission or those who are not anticipated to resume oral intake because of pre-existing swallowing problems (severe dysphagia, stroke, etc.) as they may not benefit from the nutritional intervention.

We plan to randomize family members of eligible patients. By family member, we use a broad definition of family including both relatives and close friends that know the patient and either live with or are involved in the ongoing care of the patient. They are individuals who provide support and with whom the patient has a significant relationship [1]. To be included, family members must be ≥ 18 years old, be present and expected to visit regularly (minimum about 3 times a week) while the patient is in hospital, must be the nominated or legally appointed substitute decision-maker (SDM), and be able to communicate in English (verbally and in writing). We are aiming to work with the same person(s) over the entire study period. However, if there are multiple SDMs, we will work with all that are available and interested. However, we will have them select one designated individual to fill out the evaluation questionnaires.

**References**

[1] Davidson JE, Powers K, Hedayat KM, Tieszen M, Kon AA, Shepard E, et al. Clinical practice guidelines for support of the family in the patient-centered intensive care unit: American College of Critical Care Medicine Task Force 2004-2005. Crit Care Med. 2007;35:605-22.
