# Supplementary material for: Improving partnerships with family members of ICU patients: study protocol for a randomized controlled trial
Source: Trials. 2018 Jan 4;19:3. doi: 10.1186/s13063-017-2379-4 (PMC5753514; doi:10.1186/s13063-017-2379-4)
Supplement: Supplementary file 4 — Baseline data collection. (DOCX 45 kb) [file 13063_2017_2379_MOESM4_ESM.docx]

**Additional file 4: Baseline Data Collection**

At baseline, we will record age, admission diagnosis, admission type (medical vs. surgical), source of admission, presence of advance directives, time from hospital admission to ICU admission, Acute Physiology and Chronic Health Evaluation (APACHE) II score [1], Functional Comorbidity Index [2], and the Charlson Comorbidity Index [3]. To capture patient’s baseline nutritional status, we will use the Malnutrition Screening Tool [4] and the Nutrition Risk in Critical Illness (NUTRIC) score [5]. These will all be abstracted from the medical record. To better characterize the baseline health state of enrolled patients, we will also obtain from the family member an assessment of patient frailty (using the Clinical Frailty Scale [6]), baseline activities of daily living using the PPS [7], baseline physical function [8], and history of recent hospitalizations (over the past year; to describe the patient’s health trajectory). Multiple organ dysfunction at ICU admission will be measured using sequential organ failure assessment (SOFA) scores [9]. Use of life-sustaining treatment (mechanical ventilation, vasoactive drugs, and renal replacement therapy) and length of stay in ICU and hospital will be abstracted from the chart. Medical orders for administration, withholding, or withdrawing of life-sustaining treatment before ICU admission and during ICU stay will also be recorded. We also will document the demographics of the participating family member, including their age, sex, and relationship to the patient.

**References**

[1] Knaus WA, Draper EA, Wagner DP, Zimmerman JE. APACHE II: a severity of disease classification system. Crit Care Med. 1985;13:818-29.

[2] Groll DL, To T, Bombardier C, Wright JG. The development of a comorbidity index with physical function as the outcome. J Clin Epidemiol. 2005;58:595-602.

[3] Charlson ME, Pompei P, Ales KL, MacKenzie CR. A new method of classifying prognostic comorbidity in longitudinal studies: development and validation. J Chronic Dis. 1987;40:373-83.

[4] Ferguson M, Capra S, Bauer J, Banks M. Development of a valid and reliable malnutrition screening tool for adult acute hospital patients. Nutrition. 1999;15:458-64.

[5] Heyland DK, Dhaliwal R, Jiang X, Day AG. Identifying critically ill patients who benefit the most from nutrition therapy: the development and initial validation of a novel risk assessment tool. Crit Care. 2011;15(6):R268.

[6] Bagshaw SM, Stelfox HT, McDermid RC, Rolfson DB, Tsuyuki RT, Baig N, et al. Association between frailty and short- and long-term outcomes among critically ill patients: a multicenter prospective cohort study. CMAJ. 2014;186:E95-102.

[7] Anderson F, Downing GM, Hill J, Casorso L, Lerch N. Palliative performance scale (PPS): a new tool. J Palliat Care. 1996;12:5-11.

[8] Ware JE. The SF-36 health survey. In: Spilker B, editor. Quality of Life and Pharmacoeconomics in Clinical Trials. 2^nd^ ed. Philadelphia: Lippincott-Raven Publishers; 1996. p. 337-345.

[9] Moreno R, Vincent JL, Matos R, Mendonça A, Cantraine F, Thijs L, et al. The use of maximum SOFA score to quantify organ dysfunction/failure in intensive care. Results of a prospective, multicentre study. Working Group on Sepsis related Problems of the ESICM. Intensive Care Med. 1999;25:686-96.
